# Mannose-binding lectin deficiency and NOD2 mutations do not predispose to *Staphylococcus aureus *bloodstream infections but may influence outcome

**DOI:** 10.1186/cc11759

**Published:** 2012-11-14

**Authors:** M Osthoff, HM Au Yong, MM Dean, D Eisen

**Affiliations:** 1 Royal Melbourne Hospital, Victorian Infectious Diseases Service, Parkville, Australia; 2 Australian Red Cross Blood Service, Brisbane, Australia

## Background

*Staphylococcus aureus *is a major cause of bloodstream infections (BSI), and is associated with a higher morbidity and mortality compared with other BSI pathogens. Innate pattern recognition receptors like mannose-binding lectin (MBL) of the complement system and NOD2 (nucleotide-binding oligomerization domain-containing protein 2), an intracellular sensor for a variety of pathogens, have been shown to be crucially involved in the immune response against *S. aureus *in knockout animal models [1,2], but human data are lacking. Low MBL levels and NOD2 mutations can be found in up to 30% and 10% in the general population, respectively [3,4]. This study aimed to investigate whether MBL deficiency and NOD2 mutations predispose to and influence the severity of *S. aureus *BSI.

## Methods

A matched case-control study was undertaken involving 70 patients with *S. aureus *BSI and 70 age-matched and sex-matched hospitalized controls recruited prospectively at two major tertiary hospitals. Participant blood samples were analyzed for MBL levels by mannan-binding ELISA and for four *MBL2 *and three *NOD2 *polymorphisms by real-time PCR. Clinical and microbiological data were reviewed. MBL deficiency was defined as functional MBL level ≤0.1 μg/ml. Univariate and multivariate conditional logistic regression was used to investigate the risk of BSI in matched controls and cases.

## Results

**Figure 1:**
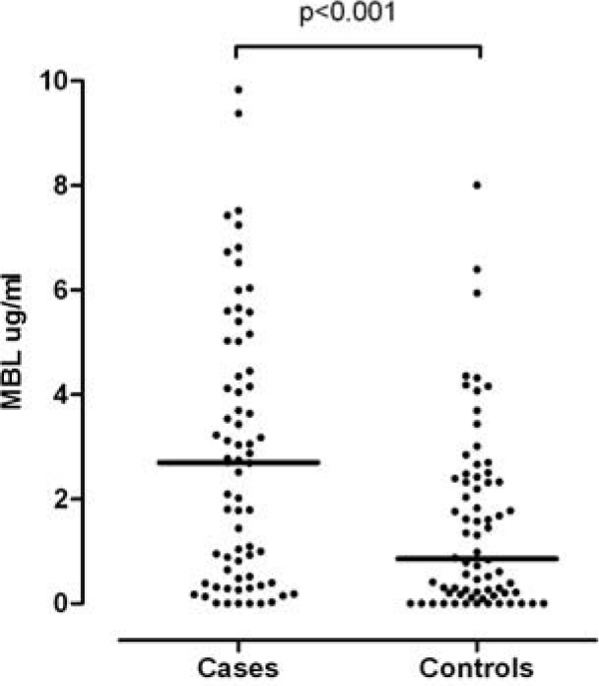
**Differences in MBL serum concentrations in cases (*S. aureus *BSI) and controls**. Horizontal lines represent medians.

*S. aureus *BSI were nosocomially acquired (60%) and intravenous catheter associated (50%) in the majority of cases with an in-hospital mortality of 10%. After adjusting for diabetes, immunosuppression, chronic kidney disease and long-term intravenous catheters, MBL deficiency was found less frequently in cases than controls (8.6% vs. 20%, OR = 0.38, *P *= 0.07) as were low-producing MBL genotypes (11% vs. 23%, OR = 0.37, *P *= 0.05), whereas NOD2 polymorphisms were similarly distributed (14% vs. 10%, *P *= 0.4). In line with *MBL2 *genotypic results, MBL levels were significantly higher in cases than in controls (adjusted OR = 1.35 per 1 μg/ml increase, *P *= 0.002; Figure 1). Cases with *NOD2 *polymorphisms had less severe disease manifestations as shown by a lower SOFA score (mean 2.1 vs. 4.4, *P *= 0.04) and a reduced rate of multiorgan dysfunction and death (40% vs. 60%, *P *= 0.06), whereas MBL deficiency had no influence on the severity of *S. aureus *BSI.

## Conclusion

Neither MBL deficiency nor *NOD2 *polymorphisms were associated with an increased risk of *S. aureus *BSI. In fact, contrary to experimental data, MBL deficiency seemed to confer protection in acquiring *S. aureus *BSI, and NOD2 mutations were less frequently associated with multiorgan dysfunction in this matched case-control study.
